# Comparison of pregnancy outcomes of letrozole-induced frozen-thawed embryo transfer cycles in PCOS women with two different abnormal ovulation patterns: A retrospective cohort study

**DOI:** 10.1097/MD.0000000000033049

**Published:** 2023-02-17

**Authors:** Dan-Dan Wang, Jing-Xian Cao, Wen-Jing Jiang, Jin-Wei Hou, Meng-Han Yan, Zhen-Gao Sun, Jing-Yan Song

**Affiliations:** a The First Clinical College, Shandong University of Traditional Chinese Medicine, Jinan, People’s Republic of China; b School of Traditional Chinese Medicine, Shandong University of Traditional Chinese Medicine, Jinan, People’s Republic of China; c Reproductive and Genetic Center of Integrated Medicine, Affiliated Hospital of Shandong University of Traditional Chinese Medicine, Jinan, People’s Republic of China.

**Keywords:** anovulation, frozen-thawed embryo transfer, infertility, oligo-ovulation, polycystic ovary syndrome

## Abstract

No studies have been conducted on the impact of different types of ovulatory dysfunction on the outcomes of frozen-thawed embryo transfers (FETs) in a letrozole-stimulated cycle in women with polycystic ovarian syndrome (PCOS). This study aimed to compare whether pregnancy outcomes of the letrozole-induced protocol in FET cycles differed between oligo-ovulatory and anovulatory women with PCOS. In a retrospective cohort study, women with PCOS who had undergone letrozole-induced FET at a university-affiliated fertility clinic from February 2014 to October 2020 were identified. The primary end point was live birth rate (LBR) per embryo transfer. Propensity score matching and multivariate logistic regression analyses were performed to control for the relevant confounders. A total of 652 women with PCOS undergoing letrozole-induced FET were included in the final analysis. Three hundred sixty-three of these women had oligo-ovulatory periods, while 289 had anovulatory periods. Propensity score matching analysis showed that LBR did not differ between groups (36.8% in oligo-ovulatory group vs 32.8% in anovulatory group, *P* = .431). Nevertheless, after controlling for potential confounding factors, LBR was significantly lower in anovulatory than oligo-ovulatory women (adjusted odds ratio 1.57, 95% confidence interval 1.08–2.29, *P* = .018). Furthermore, the pregnancy loss rate among the oligo-ovulatory group remained lower than those among the anovulatory group (adjusted odds ratio 0.23, 95% confidence interval 0.12–0.44, *P* < .001). Despite adjustment for confounding factors, those with oligo-ovulatory PCOS had a higher LBR and lower pregnancy loss rate compared with those with anovulatory PCOS. This may indicate that when oligo-ovulation is detected, PCOS patients should be intervened in time to conceive as soon as possible. Prospective studies must be conducted in the future to verify our findings.

## 1. Introduction

The freeze-all strategy has become an accepted and valuable alternative to fresh embryo transfer (ET) in assisted reproductive technology, thanks to increasing demands for fertility treatment and improved cryopreservation techniques. Generally speaking, elective frozen-thawed embryo transfer (eFET) has good outcomes similar to fresh ET and, in some circumstances, even better than fresh ET in the general population requiring fertility treatment.^[1]^ In contrast to fresh ET, frozen-thawed embryo transfer (FET) allows the embryo and endometrium to become more synchronized, reducing the risks of high-dose gonadotropin stimulation (Gn) and ovarian hyperstimulation syndrome (OHSS) and maintaining pregnancy rates similar to those achieved with fresh ET treatment.^[2]^ Nearly 90% of study participants will choose eFET, despite its delay, because it presents reduced risk to mother and child. The majority of patients are willing to undergo either eFET or freeze all strategies in the event of equal pregnancy rates, if there is significant prior information on the basic evidence of safety and risk parameters.^[3]^

Among polycystic ovarian syndrome (PCOS) patients, an excessive response to ovarian stimulation is considered to be an independent risk factor for OHSS.^[4,5]^ In addition, OHSS is caused by the supra-physiological hormone milieu resulting from ovarian stimulation, which may interfere with embryo implantation and placenta development.^[6]^ The primary goal of assisted reproductive technology is embryo implantation, which is influenced by 3 critical factors: quality of the embryo, receptivity of the endometrium, and optimal synchronization between the endometrium and embryo development.^[7]^ Thus, it is essential to determine the implantation acceptance window and do optimal endometrial preparation in FET cycles in order to achieve optimal reproductive outcomes. There are 2 common endometrial preparation strategies for FET in PCOS women: cycles where the endometrium is artificially prepared with estrogen and progesterone, commonly referred to as hormone replacement therapy FET cycles, and cycles in which ovulation is induced via drugs (ovulation induction cycles). However, the number of high-quality randomized controlled trials is small, and there is a lack of consensus on how to prepare and synchronize the endometrium.^[8–10]^

Based on the available evidence, eFET is generally more effective than fresh ET for achieving live births in women with PCOS.^[11]^ The most commonly used endometrial preparation options before FET for patients with abnormal ovulation are letrozole-induced ovulation and hormone replacement therapy cycles.^[12]^ Most specifically, in PCOS patients undergoing FET, it has been reported that compared with artificial cycles, letrozole stimulation cycles may be associated with higher live birth rate (LBR) and lower risk of miscarriage, preterm birth and preeclampsia.^[13]^ A significant increase in cycle cancelations was seen in stimulation cycles due to luteinizing hormone (LH) surges, progesterone rises, and unintended arrest of follicle development, although stimulation cycles had similar average endometrial thicknesses as artificial cycles.^[14]^ Nevertheless, letrozole stimulation protocols may be a reasonable option for endometrial preparation before FET in PCOS women. It has been reported that 16% of 316 women with PCOS had normal-appearing cycles despite their oligo-anovulatory cycles.^[15]^ In terms of the phenotypic of ovarian dysfunction of PCOS, the oligo-anovulation subgroup is generally milder than the anovulation subgroup.^[16]^ In contrast, no studies have been conducted on the impact of different types of ovulatory dysfunction on the outcomes of FETs in a letrozole-stimulated cycle in women with PCOS. In light of the increasing prevalence of freezing strategies for PCOS patients around the world, this topic needs to be explored. To determine whether pregnancy outcomes differ between oligo-ovulatory and anovulatory women, we conducted this study using the letrozole-induced endometrial preparation protocol prior to FET.

## 2. Materials and methods

### 2.1. Study design and participants

To determine eligibility, Infertile women with PCOS having their first FET following a freeze-all cycle conducted at the reproductive and genetic center of the affiliated hospital of Shandong University of Traditional Chinese Medicine between February 2014 and October 2020 were reviewed. Data were extracted from the electronic medical record system. The reproductive ethical review board of the hospital approved this retrospective cohort study. Because of the retrospective nature of this study, no individual patient consent was required.

The Chinese diagnostic criteria for PCOS propose that oligomenorrhea, amenorrhea, or irregular uterine bleeding are necessary conditions for diagnosis.^[17]^ Additionally, polycystic ovary morphology on ultrasound is required. Patients who fulfilled both criteria were included in this study. Specifically, patients with oligo-ovulation included in the study had menstrual cycles > 35 days at the same time as ≤ 8 ovulations per year. As far as anovulation, which is the absence of menses for a period of ≥ 6 months (amenorrhea). To confirm that ovulation has not occurred, we may obtain serum progesterone levels in a suspected midluteal cycle, and if levels are below 3 to 4 ng/mL, the cycle is considered anovulatory.

Patients aged > 40 years or with a serum basal follicle stimulating hormone (FSH) of ≥ 12 IU/L at oocyte retrieval, body mass index < 18.5 kg/m^2^ or > 35 kg/m^2^ at screening, previous in vitro fertilization attempts regardless of previous fresh or frozen-thawed ETs, recurrent spontaneous miscarriage (defined as ≥ 2 previous biochemical or clinical pregnancy losses), congenital uterine malformations, and any ovulation drugs prior to study enrollment were excluded. Additionally, preimplantation genetic screenings in FET cycles were excluded. Women who had multiple deliveries in the database were only able to retain the first pregnancy.

### 2.2. Ovarian stimulation and laboratory procedures

Previously, we described how ovarian stimulation protocol led to oocyte retrieval and embryo cryopreservation in “fresh” cycles at our center, which was routine.^[18,19]^ For ovarian stimulation, gonadotrophin-releasing hormone antagonists was used. At the time of vitrification, all transferred embryos had at least the 6-cell stage with no more than 20% fragmentation, and the blastocysts had an inner cell mass and trophectoderm grade of BB or better. Vitrification was performed on day 3 or 5 after oocyte retrieval and insemination, as previously described.^[20]^

### 2.3. Endometrial preparation and FET

In letrozole-induced FET cycles, letrozole tablets (2.5 mg, Hengrui Pharmaceuticals Co., Ltd., Jiangsu, China) were administered orally for 5 days, beginning on day 3 of spontaneous menstruation or progesterone-induced withdrawal bleeding, at a daily dosage of 5 mg. The ultrasound monitoring and serum endocrine hormone analysis were performed from menstrual cycle (MC) day 10 onwards. If the dominant follicle reached a diameter of ≥ 14 mm on MC day 10, transvaginal ultrasonography was repeated every 2 days and no additional drugs were administered until the ovulation trigger was achieved. In case of a dominant follicle < 14 mm on MC day 10, supplement with 75 IU of human menopausal gonadotropin (BBCA Pharmaceutical Co., Ltd., Anhui, China) daily to stimulate follicle growth, increasing the dosage by 37.5 to 75 IU if needed. When the average diameter of the dominant follicle reached ≥ 17 mm and endometrial thickness was ≥ 7 mm, with serum estradiol (E_2_) levels > 150 pg/mL and progesterone levels < 1 ng/mL, the timing of human chorionic gonadotropin (hCG) triggering depended on the occurrence of a LH surge. If a serum LH surge was detected (LH ≥ 20 IU/L and more than double the average LH level over the past 2 days), a bolus of 8000 IU urinary hCG (2000 IU, Livzon Pharmaceutical Group Inc., Guangdong, China) was injected at 3:00 _PM_ in the same afternoon and day 3 ET was scheduled 4 days later (6 days later for blastocyst transfer). Progesterone was administered vaginally (90 mg/day, 8% Crinone, Merck Serono, England) or intramuscularly (20 mg/day, Xianju Pharmaceutical Co., Ltd., Zhejiang, China) beginning 2 days after hCG injection. Without such an LH surge (LH < 20 IU/L), hCG was administered at 9:00 _PM_ and ET was scheduled 5 days later for day 3 embryos or 7 days later for blastocysts. Three days following the ovulatory trigger, progesterone exposure was commenced. The cycle would be discontinued if there was no dominant follicle after 14 days of ovarian stimulation, or if the follicle achieved maturity diameter but the endometrial thickness did not exceed 7 mm. Luteal phase support was continued to 10 weeks of gestation if a pregnancy occurred.

### 2.4. Study endpoints and definitions

We compared pregnancy outcomes in the group with oligo-ovulatory and anovulatory menstrual cycles. The primary outcome measure was LBR, defined as at least 24 weeks of gestation or at least 500 g. The secondary outcome measures were positive pregnancy rate, clinical pregnancy rate, ectopic pregnancy rate, as well as pregnancy loss rate. Serum β- human chorionic gonadotropin was measured 11 to 14 days after ET. Positive pregnancy (biochemical pregnancy), that is, serum β- human chorionic gonadotropin level ≥ 10 mIU/mL, 14 days after ET. Biochemical pregnancy loss is defined as non-visualized pregnancy losses documented only by a serum positive pregnancy test. Clinical pregnancy is defined as an intrauterine gestational sac with fetal heartbeat detected by TVUS after 7 weeks of gestation; ectopic pregnancy is defined as the development of the fertilized egg outside the uterine cavity; clinical pregnancy loss is defined as termination of pregnancy at <28 weeks of gestation with a fetus weighing <1000 g.

### 2.5. Statistical analysis

Shapiro–Wilk test was used to evaluate the normality of the data. The quantitative variables were expressed as mean ± standard deviation or median (interquartile range, interquartile range), and were analyzed by independent Student *t* test or a Mann-Whitney *U* test depending on the normality of the distribution. Qualitative variables were expressed as frequency and percentage. The χ^2^ or Fisher exact test was used to compare categorical variables. Two-tailed α of 0.05 were employed in all statistical tests. Additionally, in order to limit bias, propensity score matching (PSM) adjustment for potential confounding factors and selection bias was established. The variables included: female age, type of infertility, duration of infertility, gravidity, parity, body mass index (BMI), antral follicle count (AFC), basal FSH, basal LH, basal E_2_, AFC, Gn days, Gn dosage, oocytes retrieved, 2PN oocytes, embryos available for transfer, blastocysts available for transfer. The personal propensity score (PS) was calculated using the logit model. The oligo-ovulatory group was paired 1:1 with the anovulatory group using the nearest-neighbor random matching algorithm. The caliper width was 0.01 standard deviation of the PS. To explore potential predictors of the LBR, multivariable logistic regression analysis has been used with the LBR as the dependent variable and the type of abnormal ovulation (oligo-ovulation vs anovulation) as the primary independent variable. The potential contributing factors considered for the analysis were female age, BMI, basic FSH, basic LH, basic E_2_, initial treatment (in vitro fertilization, intracytoplasmic sperm injection), AFC, ET stage (day 3, day 5), number of embryos transferred (single, double), good-quality embryos transferred (yes, no), and endometrial thickness before FET. All of the variables were included into the logistic regression model simultaneously. The likelihood of LBR following letrozole-induced FET is represented as crude odds ratio (OR) and adjusted OR with a 95% confidence interval (CI). All analyses were performed using SPSS software version 26.0 (SPSS Inc., Chicago, IL).

## 3. Results

### 3.1. Baseline characteristics of the study population

**Figure 1.**
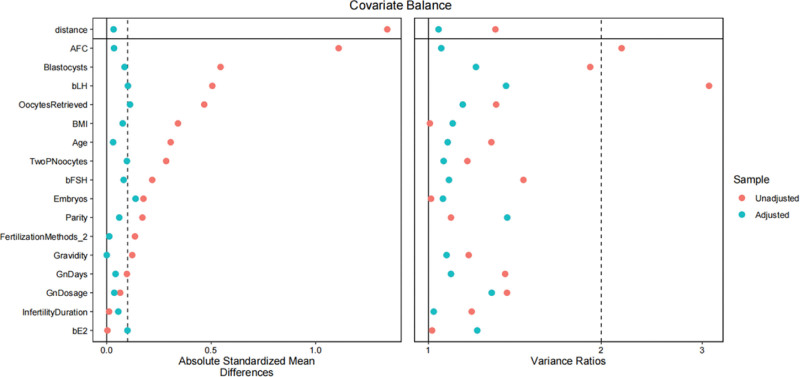
Distribution of propensity score and standardized difference before and after matching for oligo-ovulatory and anovulatory groups.

Patient profiles are shown in Table 1 before and after PSM. Overall, 652 FET cycles (363 cycles in the oligo-ovulatory group and 289 cycles in the anovulatory group) were evaluated. In each group, 174 matched pairs were identified by the PSM analysis strategy. As expected, no significant between-group differences were found in post-matching analysis with regard to all baseline characteristics, including female age, BMI, infertility type and duration, gravidity, parity, basal FSH, basal LH, basal E_2_, AFC, initial treatment, Gn days, Gn dosage, oocytes retrieved, 2PN oocytes, embryos available for transfer as well as blastocysts available for transfer. (all *P* > .05; see Table 1). The distributions of PS and standardized difference were shown in Figure 1.

**Table 1:** Demographic characteristics of 2 groups before and after PSM.

	Before PSM			After PSM		
Variables	Anovulatory group (N = 289)	Oligoovulatory group (N = 363)	*P* value	Anovulatory group (N = 174)	Oligoovulatory group (N = 174)	*P* value
Female age (years)	30.1 ± 4.3	31.6 ± 4.8	<.001*	30.8 ± 4.6	31.3 ± 4.9	.195*
BMI (kg/m^2^)	24.0 ± 3.3	22.9 ± 3.3	<.001*	23.5 ± 3.0	23.3 ± 3.1	.936*
Infertility duration (years)	3 (2,5)	3 (2,4)	.768‡	3 (2,5)	3 (2,5)	.567‡
Gravidity (n)	0 (0,1)	1 (0,2)	.159‡	1 (0,2)	1 (0,2)	.683‡
Parity (n)	0 (0,0)	0 (0,1)	.006‡	0 (0,0)	0 (0,1)	.293‡
Infertility type (n, %)			.322†			.108†
Primary infertility	153/289 (52.9%)	178/363 (49.0%)		92/174 (52.9%)	77/174 (44.3%)	
Secondary infertility	136/289 (47.1%)	185/363 (51.0%)		82/174 (47.1%)	97/174 (55.7%)	
bFSH (IU/L)	5.6 (3.9,7.1)	6.3 (4.3,7.8)	.003‡	5.9 (4.0,7.5)	6.2 (3.8,7.5)	.635‡
bLH (IU/L)	4.3 (2.3,7.0)	3.7 (2.4,5.4)	.003‡	3.8 (2.1,6.0)	3.9 (2.3,6.0)	.827‡
bE2 (IU/L)	32 (23,45)	32.1 (23,46)	.905‡	28.6 (22,41)	32.1 (22,44)	.700‡
AFC (n)	33.0 (24.5,42.5)	22 (14,29)	<.001‡	27 (19,34)	25.5 (20,35)	.644‡
Initial treatment (n, %)			.080†			.726†
IVF	205/289 (70.9%)	234/363 (64.5%)		123/174 (70.7%)	120/174 (69.0%)	
ICSI	84/289 (29.1%)	129/363 (35.5%)		51/174 (29.3%)	54/174 (31.0%)	
Gn days (n)	12.0 ± 3.2	11.8 ± 2.8	.257*	12.4 ± 2.9	11.5 ± 2.8	.700*
Gn dosage (IU)	1909.4 ± 470.1	1944.6 ± 550.2	.379*	1946.2 ± 514.7	1857.8 ± 497.2	.941*
Oocytes retrieved (n)	20 (14,26)	16 (11,22)	<.001‡	18 (13,24)	17 (12,24)	.883‡
2PN oocytes (n)	11 (7,16)	10 (6,14)	.001‡	11 (7,14)	10 (6,14.3)	.572‡
Embryos available for transfer (n)	6 (4,8)	5 (4,8)	.013‡	5 (4,8)	6 (4,8)	.937‡
Blastocysts available for transfer (n)	0 (0,1)	2 (0,3)	<.001‡	0 (0,2)	0 (0,2)	.143‡
Embryo transfer stage (n, %)			.493†			.093†
Cleavage stage (day 3)	227/289 (78.5%)	293/363 (80.7%)		129/174 (74.1%)	142/174 (81.6%)	
Blastocyst stage (day 5)	62/289 (21.5%)	70/363 (19.3%)		45/174 (25.9%)	32/174 (18.4%)	
NO. of embryo transferred (n, %)			.311†			.331†
Single embryo transfer	76/289 (19.3%)	83/363 (22.9%)		50/174 (28.7%)	42/174 (24.1%)	
Double embryo transfer	213/289 (73.7%)	280/363 (77.1%)		124/174 (71.3%)	132/174 (75.9%)	
Good-quality embryos transferred (n, %)			.984†			.162†
Yes	83/289 (28.7%)	104/363 (28.7%)		59/174 (33.9%)	47/174 (27.0%)	
No	206/289 (71.3%)	259/363 (71.3%)		115/174 (66.1%)	127/174 (73.0%)	
Endometrial thickness before FET (mm)	10.1 ± 1.8	9.8 ± 1.9	.094*	10.0 ± 1.8	10.1 ± 1.9	.132*

Data are presented as mean ± SD, n (%) or median (IQR).

2PN = Number of embryos with two pronucleus, bFSH = basic follicle stimulating hormone, BMI = body mass index, FET = frozen-thawed embryo transfer, ICSI = intracytoplasmic sperm injection, IVF = in vitro fertilization, PSM = Propensity score matching.

* *t* test for Equality of Means.

† χ^2^-test.

‡ Independent-Samples Mann–Whitney *U* test.

### 3.2. Characteristics of the FET cycles

Table 1 shows that before and after PSM, the distribution of embryos transferred, the ET stage, and good-quality embryos transferred between oligo-ovulatory and anovulatory groups were comparable (all *P* > .05). The endometrial thickness before FET was also similar across study groups (*P* > .05). Meanwhile, the anovulatory group used additional Gn for a longer period (7.3 ± 2.5 vs 5.5 ± 1.8, *P* < .001, before PSM; 6.5 ± 2.3 vs 5.4 ± 1.9, *P* = .004, after PSM) and experienced a higher cycle cancelation rate than the oligo-ovulatory group (8.7% vs 4.7%, *P* < .001, before PSM; 9.2% vs 3.4%, *P* = .028, after PSM).

### 3.3. Pregnancy and birth outcomes

In Table 2, there were no significant differences between groups in positive pregnancy rate, clinical pregnancy rate, ectopic pregnancy rate, and LBR (all *P* > .05), regardless of PSM analysis. While there was no significant difference between the biochemical and total pregnancy losses between the oligo-ovulatory and anovulatory groups following PSM (10.6% vs 7.7%, *P* = .508), the rates of clinical pregnancy loss (31.0% vs 8.3%, *P* < .001) and total pregnancy loss (38.3% vs 15.4%, *P* = .001) were significantly higher in the anovulatory group.

**Table 2:** Pregnancy outcomes of FET cycles before and after PSM.

	Before PSM			After PSM		
Outcomes	Anovulatory group (N = 289)	Oligoovulatory group (N = 363)	*P* value *	Anovulatory group (N = 174)	Oligoovulatory group (N = 174)	*P* value ^a^
Positive pregnancy rate (per transfer)	152/289 (52.6%)	165/363 (45.5%)	.070	94/174 (54.0%)	78/174 (44.8%)	.086
Clinical pregnancy rate (per transfer)	130/289 (45.0%)	154/363 (42.4%)	.513	84/174 (48.3%)	72/174 (41.4%)	.196
Biochemical Pregnancy loss rate (per positive pregnancy)	22/152 (14.5%)	11/165 (6.7%)	.087	10/94 (10.6%)	6/78 (7.7%)	.508
Clinical pregnancy loss rate (per clinical pregnancy)	36/130 (27.7%)	12/154 (7.8%)	<.001	26/84 (31.0%)	6/72 (8.3%)	<.001
Total pregnancy loss rate (per positive pregnancy)	58/152 (38.2%)	23/165 (13.9%)	<.001	36/94 (38.3%)	12/78 (15.4%)	.001
Ectopic pregnancy rate (per positive pregnancy)	2/152 (1.3%)	3/165 (1.8%)	>.999	1/94 (1.1%)	2/78 (2.6%)	.589
Live birth rate (per transfer)	92/289 (31.8%)	139/363 (38.3%)	.087	57/174 (32.8%)	64/174 (36.8%)	.431

Data are presented as n (%).

FET = frozen-thawed embryo transfer, PSM = propensity score matching.

* χ^2^-test.

### 3.4. Multivariable regression of LBR

Likewise, we used a binary multi-factor logistic regression model to examine the association between abnormal ovulatory patterns among PCOS women and pregnancy outcomes, adjusted for potential confounders (Table 3). In the adjusted models, anovulation was associated with an increased likelihood of pregnancy loss (OR 0.23, 95% CI (0.12–0.44), *P* < .001) and a decreased likelihood of live birth compared with oligo-ovulation (OR 1.57, 95% CI (1.08–2.29), *P* = .018).

**Table 3:** Relationship between endometrial preparation and pregnancy outcomes in different models.

		Crude model *		Adjusted model †	
Pregnancy outcomes	Endometrial preparation	OR (95% CI)	*P* value	OR (95% CI)	*P* value
Positive pregnancy	Anovulatory group	Reference		Reference	
	Oligoovulatory group	0.75 (0.55–1.02)	0.070	0.89 (0.62–1.27)	0.516
Clinical pregnancy	Anovulatory group	Reference		Reference	
	Oligoovulatory group	0.90 (0.66–1.23)	0.513	1.04 (0.73–1.49)	0.826
Pregnancy loss	Anovulatory group	Reference		Reference	
	Oligoovulatory group	0.26 (0.15–0.45)	<0.001	0.23 (0.12–0.44)	<0.001
Live birth	Anovulatory group	Reference		Reference	
	Oligoovulatory group	1.33 (0.96–1.84)	0.087	1.57 (1.08–2.29)	0.018

CI = confidence interval, OR = odds ratio.

* No adjustments for other covariates.

† Adjusted for female age, BMI, basic FSH, basic LH, basic E_2_, initial treatment (IVF, ICSI), AFC, embryo transfer stage (day 3, day 5), number of embryos transferred (single, double), good-quality embryos transferred (yes, no) and endometrial thickness before FET.

## 4. Discussion

To our knowledge, this is the first study to evaluate the impact of 2 ovulatory disorder patterns, oligo- and anovulation, on pregnancy outcomes in a letrozole-induced FET cycle in women with PCOS. Hatoum et al^[21]^, emphasized the benefits of more “natural” hormone support through the use of physiological endometrial preparation protocols in oligo-ovulatory or anovulatory women leading to luteal development during stimulation cycles, which may have advantages in reproductive outcomes of FET cycles compared with artificial cycles. The stimulation cycle seems to be related to higher LBR, lower pregnancy loss rate (PLR) and reducing the risk of pregnancy induced hypertension.^[12]^ However, there is little literature on the difference of pregnancy outcomes between oligo-ovulation and anovulation undergoing letrozole-induced FET cycles in PCOS women. When compared to the anovulatory group, the oligo-ovulatory group was related with a higher likelihood of a live birth and a decreased risk of miscarriage, according to the present study.

Polycystic ovarian syndrome, which is considered to be the most common endocrine disease, affects 5% to 20% of women of reproductive age worldwide.^[22]^ The pathogenesis of PCOS is complex and not entirely understood. In most cases, PCOS is diagnosed at an infertility clinic when women present with oligo- or anovulation. In comparison with PCOS women with anovulatory phenotypes, these oligo-ovulatory women appear to have a different metabolic and hormonal profile.^[23,24]^ The oligo-ovulatory woman may exhibit milder symptoms and a less severe phenotype than anovulatory women, namely lower serum testosterone, androstenedione, and free androgen index.^[16]^ Patients with anovulatory disorders tend to be hyperandrogenic, so they are less sensitive to endogenous FSH.^[25]^ There may be an explanation for the higher cycle cancelation rates in the anovulatory group and the longer additional Gn use time.

Specifically, we found that anovulation in PCOS was associated with higher PLRs and lower LBRs. Ovarian granulosa cells (GCs) form gap junctions with oocytes and play an essential role in the growth and development of oocytes.^[26,27]^ PCOS development has been established as a product of aberrant ovarian GCs proliferation and apoptosis.^[28,29]^ The dysfunction of lipid and fatty acid metabolism will damage the physiological function of ovarian GCs, resulting in the interruption of oocyte maturation and ovulation.^[30]^ Additionally, androgen exposure significantly affects the metabolic state of GCs in PCOS women, and impairs follicular growth and oocyte maturation, while the potential molecular mechanism remains to be explored in the future.^[31]^ Moreover, the endocrine and ovulatory disturbances observed in PCOS exist on a continuum, with a milder phenotype of elevated androgen and follicles evident in eumenorrheic women with oligo-ovulation, which is indicative of factors that exist along a continuum of testosterone levels, including those without clinical hyperandrogenism (HA).^[32]^ In parallel, a proof-of-concept study proposes that menstrual shedding may improve endometrial progesterone sensitivity, general endometrial function, and, possibly, pregnancy outcomes, in women with PCOS.^[33]^ On the other hand, amenorrhea, as well as HA, are closely related to pregnancy complications in PCOS women, whereas the risk is not significant in women with PCOS without HA or with normal ovulatory cycles.^[34]^ Therefore, the anovulation or amenorrhea may have an adverse impact on pregnancy outcomes of PCOS women, which needs further studies to verify.^[35]^

Our study has limitations. This study is a single center, retrospective study in China, which may limit the universality of our results to other ethnic groups. At the same time, we are unable to adjust their habits such as diet, exercise and smoking. Finally, indicators of metabolic disorders in both groups were also not detected. While this study is limited, we acknowledge its value. Previous relevant studies have shown that anovulatory patients with PCOS have a more severe phenotype, so this study may have some clinical relevance. The result suggests that PCOS patients should start early intervention for pregnancy when they exhibit signs of oligo-ovulation.

## 5. Conclusion

PCOS women may experience impact of varying degrees on pregnancy outcomes depending on their ovulation disorder patterns. When confounding factors were accounted for, those with oligo-ovulation who underwent letrozole-induced FET had higher LBRs and lower PLRs than those with anovulation. This may indicate that when oligo-ovulation is detected, PCOS patients should be intervened in time to conceive as soon as possible. More prospective studies must be conducted in the future to verify our findings.

## Acknowledgments

The authors thank the Reproductive and Genetic Center of Integrated Traditional Chinese and Western Medicine, Affiliated Hospital of Shandong University of Traditional Chinese Medicine for patient information, Professor Zhen-Gao Sun for his valuable support and help in statistical analysis of this study.

## Author contributions

**Conceptualization:** Jing-Yan Song, Zhen-Gao Sun.

**Data curation:** Jing-Xian Cao, Wen-Jing Jiang, Jin-Wei Hou, Meng-Han Yan.

**Methodology:** Jing-Yan Song.

**Supervision:** Zhen-Gao Sun, Jing-Yan Song.

**Writing – original draft:** Dan-Dan Wang.
